# (1,3)-β-D-glucan-based diagnosis of invasive *Candida* infection versus culture-based diagnosis in patients with sepsis and with an increased risk of invasive *Candida* infection (CandiSep): study protocol for a randomized controlled trial

**DOI:** 10.1186/s13063-018-2868-0

**Published:** 2018-09-04

**Authors:** Frank Bloos, Jürgen Held, Peter Schlattmann, Nicole Brillinger, Oliver Kurzai, Oliver A. Cornely, Daniel Thomas-Rüddel

**Affiliations:** 1 Center for Sepsis Control & Care, Jena University Hospital, Jena, Germany; 2 Department of Anesthesiology and Intensive Care Medicine, Jena University Hospital, Am Klinikum 1, 07747 Jena, Germany; 3 Mikrobiologisches Institut, Universitätsklinik Erlangen und Friedrich-Alexander-Universität (FAU) Erlangen-Nürnberg, Erlangen, Germany; 4 Institute of Medical Statistics, Computer Sciences and Documentation, Jena University Hospital, Jena, Germany; 5 Center for Clinical Studies, Jena University Hospital, Jena, Germany; 6 German National Reference Center for Invasive Fungal Infections NRZMyk, Leibniz Institute for Natural Product Research and Infection Biology, Hans-Knöll-Institute, Jena, Germany; 7 Institute for Hygiene and Microbiology, University of Würzburg, Würzburg,, Germany; 8 Cologne Excellence Cluster on Cellular Stress Responses in Aging-Associated Diseases (CECAD), Department I of Internal Medicine, University Hospital of Cologne, German Centre for Infection Research (DZIF), Cologne, Germany

**Keywords:** Sepsis, Septic shock, Invasive *Candida* infection, Early antifungal therapy, (1,3)-β-D-glucan, Biomarker

## Abstract

**Background:**

The time to diagnosis of invasive *Candida* infection (ICI) is often too long to initiate timely antifungal therapy in patients with sepsis. Elevated serum (1,3)-β-D-glucan (BDG) concentrations have a high diagnostic sensitivity for detecting ICI. However, the clinical significance of elevated BDG concentrations is unclear in critically ill patients. The goal of this study is to investigate whether measurement of BDG in patients with sepsis and a high risk for ICI can be used to decrease the time to empiric antifungal therapy and thus, increase survival.

**Methods/design:**

This prospective multicenter open randomized controlled trial is being conducted in 19 German intensive care units. All adult patients with severe sepsis or septic shock and an increased risk for ICI are eligible for enrolment. Risk factors are total parenteral nutrition, previous abdominal surgery, previous antimicrobial therapy, and renal replacement therapy. Patients with proven ICI or those already treated with systemic antifungal substances are excluded. Patients are allocated to a BDG or standard care group. The standard care group receives targeted antifungal therapy as necessary. In the BDG group, BDG serum samples are taken after randomization and 24 h later. Antifungal therapy is initiated if BDG is ≥80 pg/ml in at least one sample. We plan to enroll 312 patients. The primary outcome is 28-day mortality. Other outcomes include antifungal-free survival within 28 days after enrolment, time to antifungal therapy, and the diagnostic performance of BDG compared to other laboratory tests for early ICI diagnosis. The statistical analysis will be performed according to the intent-to-treat principle.

**Discussion:**

Because of the high risk of death, American guidelines recommend empiric antifungal therapy in sepsis patients with a high risk of ICI despite the limited evidence for such a recommendation. In contrast, empiric antifungal therapy is not recommended by European guidelines. BDG may offer a way out of this dilemma since BDG potentially identifies patients in need of early antifungals. However, the evidence for such an approach is inconclusive. This clinical study will generate solid evidence for health-care providers and authors of guidelines for the use of BDG in critically ill patients.

**Trial registration:**

Clinicaltrials.gov, NCT02734550. Registered 12 April 2016.

## Background

Invasive *Candida* infection (ICI) is a rising problem in critically ill patients. ICI incidence has increased in hospitalized patients since the beginning of this millennium [1, 2]. *Candida albicans* alone was assumed to be involved in 13% of all infections acquired in an intensive care unit (ICU) [3]. In addition, the presence of ICI is associated with a high risk of death with an attributable mortality of up to 49% [4].

The most frequent clinical representation of ICI is candidemia. The gold standard diagnostic test for candidemia is the detection of *Candida* spp. in a blood culture. Modern blood culture systems, however, detect less than 60% of all cases of ICI [5]. Furthermore, the detection and identification of *Candida* spp. with a blood culture usually takes several days. Such a diagnostic and thus therapeutic delay substantially increases mortality [6, 7].

Antifungal therapy and source control are the core elements of treating ICI. Targeted therapy in proven ICI is always the right treatment option. Due to the low diagnostic sensitivity of blood culture analysis and the high risk of death in unrecognized ICI, the addition of an antifungal agent to the empiric antimicrobial therapy for sepsis needs to be discussed. American guidelines strongly recommend empiric antifungal therapy in patients with septic shock and with risk factors for ICI [8]. However, the authors point out that the evidence for this recommendation is low and that the most important combination of risk factors in an individual patient has not been established. Furthermore, widespread use of antifungal agents must be balanced against the cost, the risk of toxicity, and the emergence of resistance [8]. Likewise, the Surviving Sepsis Campaign recommends empiric antifungal therapy in sepsis patients if the risk of ICI is sufficiently high. However, the Surviving Sepsis Campaign does not further specify the risk factors qualifying for antifungal therapy [9]. In contrast, the European guidelines recommend only a targeted antifungal therapy, since the lack of data do not support a recommendation for empiric therapy [10].

A faster diagnosis of ICI might be facilitated by biomarkers. (1,3)-β-D-glucan (BDG) is a cell wall constituent of *Candida* spp. and other fungi. The assays for BDG measurement use a modification of the limulus-amebocyte-lysate pathway. BDG activates factor G, which in turn activates a coagulation enzyme resulting in the cleavage of p-nitroaniline from a peptide substrate. The photometrically measured p-nitroaniline release over time is used to determine the BDG concentration [11]. The diagnostic accuracy of the BDG measurement has been examined in a meta-analysis as well as in a large multicenter study. BDG was able to distinguish proven or probable ICIs from no ICIs in several patient populations [12, 13]. However, the statistical heterogeneity of the available studies is large [13]. In addition, falsely increased BDG serum concentrations in the absence of ICI may be induced by several common ICU interventions. For example, concurrent bacteremia, hemodialysis membranes, administrations of blood products, and treatment with albumin or immunoglobulin products can interfere with BDG measurements [11, 14, 15]. If relevant, this type of interference would significantly reduce the clinical usability of BDG assays in critically ill patients. Current guidelines hesitate in giving a general recommendation for initiating antifungal therapy relying on BDG results [8, 9, 16].

The high risk of death caused by a delayed initiation of antifungal therapy could be addressed by a preemptive approach where starting antifungal therapy is based on early diagnostic tests such as polymerase chain reaction (PCR) or BDG. However, clinical studies addressing a preemptive antifungal therapy are not conclusive [17, 18]. Clinical guidelines are reluctant in recommending such an approach as robust data are missing [8–10].

We are undertaking this trial since there is strong evidence that early antifungal therapy in critically ill patients with ICI increases survival. However, there is missing evidence that BDG can successfully identify those critically ill patients who may profit from early antifungal therapy. The goal of this trial is to investigate whether the measurement of BDG can decrease the time to empiric antifungal therapy and increase survival in patients with severe sepsis or septic shock and an increased likelihood of ICI.

## Methods/design

CandiSep is an investigator-initiated prospective, multicenter, randomized, open, and parallel group study comparing mortality for a BDG-driven antifungal therapy versus standard care during 28 days after enrolment in adult patients with severe sepsis or septic shock and with a high risk for ICI. The study currently involves 14 ICUs in German hospitals. A further five ICUs are awaiting approval for the trial.

### Ethics

The Friedrich Schiller University Jena is the sponsor of the trial. The trial was approved by the ethics committee of the Jena University Hospital on 19 July 2016 and the German Health Authorities (BfArM) on 17 June 2016. In addition, the local ethics committees at each site approved the study protocol and the study competence of each site. Written informed consent is obtained from all patients or their legal representatives. If this is not possible before enrolment in due time, the ethics committees has approved a deferred consent process where the inability to provide consent is confirmed by an independent physician and the patient is enrolled without informed consent. As soon as the legal representative of the patient is available, written informed consent is immediately obtained; otherwise, the patient is withdrawn from the study and all study procedures are ended.

### Aims

The primary aim of the CandiSep study is to evaluate the impact of BDG-driven antifungal therapy on the rate of death from any cause by 28 days after inclusion.

The secondary objectives are to evaluate the impact of BDG in patients with sepsis on

- antifungal-free survival within 28 days after enrolment
- *Candida* colonization according to the *Candida* colonization index (CCI)
- time to antifungal therapy
- duration of organ support, including ventilation, vasopressor, and renal replacement, until day 28
- mean total score for sequential organ failure assessment (SOFA) calculated as the sum of daily SOFA scores divided by the number of study days on ICU but not longer than 14 days [19]
- ICU and length of hospital stay
- ICU and hospital mortality
- Frequency of adverse and severe adverse events

Further secondary objectives are to compare

- the diagnostic performance of BDG with other laboratory techniques to diagnose ICI early, such as PCR, mannan antigen and anti-mannan antibodies, *Candida* germ tube antibodies, and other antigen or antibody tests
- costs of antifungal therapy between the groups

### Patient population

All patients treated in the ICUs of participating hospitals are eligible for inclusion if they fulfill all the inclusion criteria and none of the exclusion criteria. Patients are screened daily by the study personnel at each study site.

#### Inclusion criteria

- Presence of severe sepsis or septic shock. Sepsis definitions were reported previously [20]. However, in the light of the Sepsis-3 definitions [21], we do not include the systemic inflammatory response syndrome (SIRS) as a requirement for sepsis diagnosis. Thus, severe sepsis in this trial was defined as microbiologically proven or clinically suspected infection combined with acute organ dysfunction (Table 1). Septic shock was defined as infection in combination with arterial hypotension or need for vasopressor therapy despite adequate fluid resuscitation.
- Onset of sepsis: ≤12 h before randomization (until 25 January 2018) or ≤24 h before randomization (since 26 January 2018, after an amendment to the study protocol)
- Increased risk of ICI with at least one of the following criteria:
  - Total parenteral nutrition defined as intravenous infusion of at least 500 ml of 20% glucose per day, maximum of 500 ml enteral feeding per day, no oral feeding other than tea or water
  - Abdominal surgery within the last 7 days
  - Antimicrobial therapy ≥48 h within the last 7 days
  - Continuous need for renal replacement therapy for chronic renal failure or renal replacement therapy for acute renal failure ≥48 h before onset of sepsis
- Informed consent of patient or their legal representative or if not possible a statement by an independent physician

**Table 1:** Definitions of organ failure

• Encephalopathy (reduced vigilance, restlessness, disorientation, or delirium without influence of psychotropic substances) • Thrombocytopenia (thrombocyte count ≤100,000/μl *or* decrease of thrombocytes > 30% in 24 h without evidence of bleeding) • Arterial hypoxemia [paO_2_ < 10 kPa (75 mmHg) when breathing room air or paO_2_/FiO_2_ ≤ 33 kPa (250 mmHg) not caused by pulmonary or cardiac disorder] • Arterial hypotension (systolic blood pressure ≤ 90 mmHg or mean blood pressure ≤ 70 mmHg) for at least 1 h despite adequate fluid resuscitation and absence of other types of shock • Renal dysfunction (urinary output ≤0.5 ml kg^-1^ h^-1^ for at least 1 h despite sufficient fluid resuscitation *or* increase of serum creatinine 2× above the reference range) • Metabolic acidosis (base deficit ≥5.0 mEq/l or serum lactate concentration 1.5 above the reference range) *Septic shock* • Diagnosis of infection Arterial hypotension (systolic blood pressure ≤ 90 mmHg or mean blood pressure ≤ 70 mmHg) for at least 2 h despite adequate fluid resuscitation which requires the administration of vasopressors (dopamine ≥5 μg kg^− 1^ min^− 1^; norepinephrine or epinephrine ≥0.05 μg kg^− 1^ min^− 1^; phenylephrine or vasopressin in any dosage) to maintain systolic blood pressure 90 mmHg or mean systolic pressure 70 mmHg	

#### Exclusion criteria

- Pregnant or lactating women
- Proven ICI as defined by the EORTC/MSG-Consensus Group [22]
- Ongoing or immediately planned systemic antifungal therapy
- Liver cirrhosis CHILD-Pugh class C
- Surgery with cardiopulmonary bypass within the last 4 weeks
- Treatment with immunoglobulins within the last 4 weeks
- Immunosuppression (e.g., for solid organ transplantation, AIDS, or leukopenia)
- Participation in another clinical study
- Previous participation in this study
- No commitment to full patient support (i.e., do not resuscitate order)
- Patient’s death is considered imminent due to coexisting disease
- Relationship to study team member (i.e. colleague, relative, or employee)

#### Trial management

The study is run by the publicly funded Center for Sepsis Control & Care. The steering committee consists of two intensivists (FB and DTR) and an external expert on fungal infections (OAC). The steering committee is supported by the Center for Clinical Studies of Jena University Hospital, which is responsible for trial management and monitoring the source data. Adherence to the study protocol is ensured by risk-based monitoring. The monitor visits each center three times during the study. The first visit occurs after the first recruitment. In addition, electronic case report forms (eCRFs) are regularly checked by the monitor and the data manager. Adverse and severe adverse events are reported until day 14 or the end of the ICU stay (whatever comes first). Any adverse event not recovered until day 28 is also reported as a severe adverse event. Adverse events are recorded in the eCRF. Severe adverse events are submitted to the trial management via fax within one working day and are assessed by an additional assessor. Severe adverse events related to study procedures or the investigational produce are forwarded to the German authorities.

The data and safety monitoring board (DSMB) is composed of three external experts (an infectious disease physician, a microbiologist, and a statistician). The DSMB is regulated by a standardized operating procedure. The main function of the DSMB is to monitor the safety of the study procedure. Thus, the DSMB receives unblinded quarterly safety reports, which are required under German legislation. The safety report contains descriptive data about serious adverse events. In addition, the DSMB evaluates annually the safety and quality of the ongoing trial by receiving notifications of adverse events, recruitment rates, and protocol violations especially regarding the treatment algorithm of the BDG group. The DSMB advises the sponsor on whether to continue or discontinue the study and on protocol amendments.

#### Randomization and study procedures

The trial sites have access to a web-based central randomization service, which is available 24 × 7. The randomization list is prepared by an independent statistician via a computer-based algorithm and is stratified by study center. Patients are randomized to one of the two study arms (control group or BDG group) in a 1:1 ratio. A unique patient ID is generated for data collection throughout the trial.

**Fig. 1:**
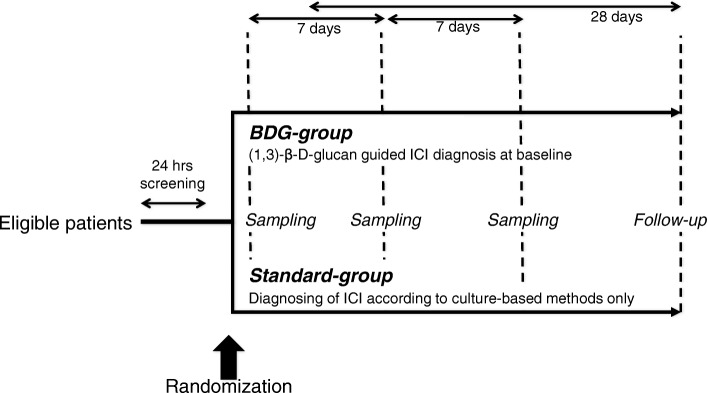
Study flow chart. Microbiological samples were taken from multiple anatomic sites to assess *Candida* colonization. BDG (1,3)-β-D-glucan, ICI invasive *Candida* infection

**Fig. 2:**
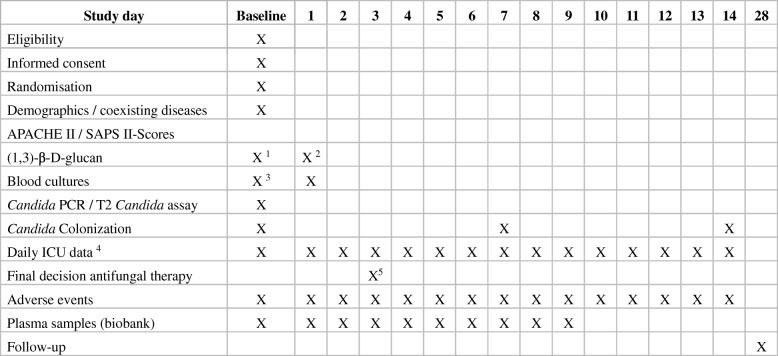
Study procedures and assessments. *ICU* intensive care unit, *SOFA* sequential organ failure assessment; 1 maximum of 1 h after randomization; 2 22–26 h after randomization; 3 ≤6 h before and maximum of 3 h after randomization; 4 Sepsis criteria, routine biochemistry, microbiological results, medication, anti-infectious measures, and SOFA score; 5 As soon as all baseline microbiological results are available

The time flow of the study is shown in Fig. 1 and a time frame of the study procedures are described in Fig. 2. Serum samples for BDG measurements are taken under standardized conditions not later than 1 h after randomization and after 24 h. Samples are left to coagulate for 30 min at room temperature and are then centrifuged at 2000*g* at 20 °C for 10 min. Serum is filled into BDG-free tubes without using a pipette and refrigerated together with an EDTA blood sample at 2–8 °C. Serum and EDTA samples are shipped at 2–8 °C via courier to the Microbiology Institute of the University Hospital Erlangen. There, BDG serum concentrations are measured using the Fungitell® assay (Associates of Cape Cod Inc., East Falmouth, MA, USA) following the standard operating procedures. Any remaining serum is frozen at −80 °C for later analysis of mannan antigen, anti-mannan antibodies, and *Candida* germ tube antibodies. EDTA blood samples are frozen at −80 °C for later measurement in additional antigen and antibody assays.

Blood cultures must be obtained by sterile venipuncture at most 3 h after randomization if no blood cultures were taken up to 6 h before randomization. An additional sample for *Candida* PCR is obtained via the same venipuncture. In addition, microbiological samples are taken from the nose or throat, skin (axillar region), rectum or feces, urine, and tracheal or bronchial secretion to determine the CCI [23]. Blood cultures are repeated on the day after randomization and microbiological samples for the CCI are repeated on day 7 and day 14 if the patient is still in the ICU.

*Candida* PCR is performed in the National Reference Center for Invasive Mycoses (NRZMyk, Hans-Knöll-Institute, Jena, Germany). EDTA samples are shipped via the regular postal service at room temperature. *Candida* spp. are detected by a seminested PCR assay amplifying the internal transcribed spacer region 2 (ITS2). Total DNA is extracted from a minimum of 3 ml of EDTA anticoagulated blood [24]. The primary PCR amplifies the entire ITS using the panfungal primers ITS1fkyo2 [25] and ITS4 [26] while for the seminested PCR, the *Candida* specific primer Cand F [27] and the panfungal primer ITS4 are used to amplify specifically the ITS2 region of *Candida* spp. PCR products are detected in agarose gels using GelRed. Species identification is achieved by sequence comparison using in-house ITS alignments including sequences of ex-type and reference strains of all clinically relevant *Candida* spp. and their sibling species. During the assay, appropriate controls for DNA extraction (negative control) and amplification (negative and positive control) are tested in parallel. PCR results are not reported to the treating physicians.

EDTA and heparin plasma samples are taken daily until day 9 after randomization or the day of ICU discharge, whichever occurs first. Samples are centrifuged at standardized conditions (2000*g* at 20 °C for 10 min) and are stored locally at −20 °C. Samples are shipped regularly on dry ice via courier to the Integrated Biobank at the Jena University Hospital where samples are stored at −80 °C.

### Control group

Serum for measurement of BDG concentration is obtained not later than 1 h after randomization and after 24 h. Samples are shipped regularly as convenient. The treating physicians are blinded for the BDG results. Treatment of ICI occurs according to European guidelines based on microbiological findings [10].

### BDG group

Serum for measurement of BDG concentration is obtained no later than 1 h after randomization and after 24 h. Each sample is shipped to the central laboratory via courier as soon as possible after the sample was obtained. Specific courier schedules depending on the distance to the central laboratory have been developed for each center. These schedules ensure that the result of the first BDG sample is available no later than 24 h after sampling. The treating physicians are informed about the BDG results via telephone and fax. Table 2 describes the therapeutic procedure depending on the BDG results. Briefly, ICI is unlikely if BDG concentrations are less than 80 pg/ml. In this case, antifungal therapy is not required. Any BDG serum concentration of ≥80 pg/ml is compatible with ICI and should be followed by initiation of antifungal therapy, which follows European guidelines [10]. Any blood culture, biopsy, or sample from physiologically sterile body fluids that are positive for *Candida* spp. are treated independently of the BDG result. The final decision about antifungal therapy is made when all microbiological results from randomization are available using a predefined treatment algorithm (Table 2). If culture results are negative for *Candida spp.*, an initially started empirical antifungal therapy is continued only if serum BDG concentrations are above 80 pg/ml in both samples.

**Table 2:** Antifungal therapy depending on (1,3)-β-D-glucan concentrations

Diagnostic results	Recommendation
(1,3)-β-D-glucan < 80 pg/ml in both measurements	No initial antifungal therapy
• No *Candida* spp. in blood culture or other primary sterile body fluids	No antifungal therapy
• *Candida* spp. proven in blood culture or other primary sterile body fluids	Start antifungal therapy according to European guidelines
(1,3)-β-D-glucan ≥ 80 pg/ml in one of two measurements	Immediately start antifungal therapy for ICI according to European guidelines [16]
• No *Candida* spp. in blood culture or other primary sterile body fluids	Antifungal therapy is discontinued
• *Candida* spp. proven in blood culture or other primary sterile body fluids	Antifungal therapy is continued
(1,3)-β-D-glucan ≥ 80 pg/ml in both measurements	Immediately start antifungal therapy for ICI according to European guidelines [16]
• No *Candida* spp. in blood culture or other primary sterile body fluids	Antifungal therapy is continued
• *Candida* spp. proven in blood culture or other primary sterile body fluids	Antifungal therapy is continued

Treatment recommendations for antifungal therapy depend on the results of the (1,3)-β-D-glucan results as well as the results from initial blood cultures and optional microbiological results from primary sterile body fluids

*ICI* invasive *Candida* infection

### Patient withdrawal

Patients are withdrawn from the study if the patient or the patient’s legal representative withdraws informed consent. In this case, all study interventions are stopped. All data captured until this time point are kept in the database and safety-related data are documented until day 28 if possible. Data from such patients become only part of the safety analysis dataset. Withdrawn patients are replaced by an additionally randomized patient with the same group assignment.

### Data collection

Data are collected via web-based data capture software (OpenClinica®) compliant with good clinical practice requirements. Data are pseudonymized. Only personnel at the study sites have access to the personalized data. Visits and study assessments are shown in Fig. 2. Assessments are made at enrolment (day 0) and for the following 14 days if the patient is still in the ICU. Assessments include parameters to calculate the SOFA score, applied supportive therapy, daily blood chemistry, microbiological samples, antimicrobial therapy as well as adverse and serious adverse events according to German legislation. Adverse and serious adverse events are reported until day 14 after randomization or when the patient is discharged from the ICU, whichever comes first. Patients or relatives are contacted to obtain survival status on day 28 after randomization. We also obtain the duration of antifungal therapy, vasopressor support, mechanical ventilation, and renal replacement therapy.

### Sample size and power

This study is designed to reject the null hypothesis that 28-day mortality is equal in the BDG control and in the control group. Studies have shown that 16.8% of patients with a CCI of 3 or more develop ICI [28]. However, it is estimated that only 60% of ICI can be proven in microbiological cultures [5]. We, therefore, estimated that 28% of the high-risk population in this study are developing ICI. In the control group, 97.6% of those patients with ICI are expected to die, as the antifungal therapy is delayed until there is microbiological proof of fungal infection [7]. It is hypothesized that early antifungal therapy triggered by BDG serum concentration will reduce ICI occurrence to 14% [29] and mortality to 52.8% [7]. The mortality of patients with a high risk for ICI without actually having ICI is estimated to be 31.2% in both groups [30].

Based on these estimates, the control group has an expected mortality of 49.8% (= 0.28 × 0.976 [mortality of patients having ICI] + 0.72 × 0.312 [mortality of patients not having ICI]) compared to 34.2% (= 0.14 × 0.528 [mortality of patients having ICI] + 0.86 × 0.312 [mortality of patients not having ICI]) in the BDG group. A two-group chi-squared test can detect this difference with alpha = 0.05 with a statistical power of 0.8 in a sample of 156 patients per group. Studies with a similar patient population have demonstrated a 10% drop-out rate [20, 31]. We, therefore, aim to randomize 348 patients to achieve 312 evaluable patients.

#### Statistical analysis

The study objectives are analyzed in the intention-to-treat population. The primary objective is analyzed with the chi-squared test. Relative risk, risk difference, and number needed to treat are reported with 95% confidence intervals. Secondary objectives are analyzed according to their scales. A chi-squared or Fisher’s exact test is applied for 28-day antifungal-free survival, *Candida* colonization, and ICU and hospital mortality. A *t*-test for independent samples or a Mann–Whitney U-test is applied for the CCI, mean total SOFA score, and organ-support-free days. Kaplan–Meier estimates and log rank tests are applied for time to antifungal therapy, and time to hospital or ICU discharge.

For the following predefined subgroups, 28- day mortality, time to antifungal therapy, and length of hospital stay are separately analyzed:

- septic shock at randomization
- CCI ≥ 0.5
- *Candida* PCR positive
- blood culture positive for *Candida* spp.
- BDG serum concentration ≥ 80 pg/ml in both samples
- more than two risk factors for ICI as defined in the inclusion criteria

The diagnostic accuracy of BDG, PCR, and other experimental tests is assessed by calculating sensitivity and specificity together with 95% confidence intervals. ICI is defined as the presence of *Candida* spp. in a tissue biopsy, in a blood culture, or in a primary sterile bloody fluid. Pharmacoeconomics are assessed by calculating the costs of antifungal therapy for each individual patient and adding the cost of BDG measurement for patients in the BDG group.

Moreover, 28-day mortality, time to antifungal therapy, and length of hospital stay are also analyzed in a per-protocol analysis. For the per-protocol analysis, patients in the BDG group are excluded if antifungal therapy did not comply with the algorithm defined in the protocol (Table 2) and patients in the control group are excluded if they received systemic antifungal therapy in the absence of proven ICI.

## Discussion

European guidelines recommend antifungal therapy only in cases where *Candida* spp. are detected in physiologically sterile body fluids [10]. If *Candida* is the underlying pathogen and treated upon microbiological proof only, the mortality of patients with septic shock is extremely high [7]. Because of this high mortality, American guidelines recommend empiric antifungal therapy in patients with a high risk of ICI [8, 9] although evidence for such a recommendation is low and risk factors have not been established. This uncertainty triggers the prescription of antifungals to critically ill patients, which is often not compliant with guidelines [32]. Thus, fast identification of ICI is an urgent need. This demand may be met by measuring BDG serum concentrations. Elevated BDG serum concentrations of more than 80 pg/ml have a high diagnostic accuracy to predict ICI [12, 13, 18, 33, 34]. However, the available data suggest that many ICU interventions may interfere by increasing BDG serum concentrations [11, 14, 15]. In addition, the diagnostic accuracy of BDG has been reported only for candidemia rather than ICI in general and may also depend on the fungal species [12]. These limitations may further affect the clinical usability of BDG. Indeed, the latest Surviving Sepsis Campaign guideline refrained from using BDG serum concentrations to indicate antifungal therapy [9]. Taken together, BDG has the potential to add significant information about *Candida* spp. as the underlying pathogen in patients with sepsis but conclusive evidence for critically ill patients is missing. As a consequence, the role of BDG remains undefined and BDG measurement is currently not part of the diagnostic work-up of infection in most ICUs.

The trial attempts to enroll patients with a high risk of ICI to increase the chance of including patients who may benefit from the study intervention. Many risk factors for developing ICI have been published [35], making it difficult to identify patients truly at risk when using all the available risk factors. Firstly, we applied elements of the CCI (total parenteral nutrition and past surgery) as risk factors. An elevated CCI is associated with a high incidence of ICI [28]. Secondly, we performed a meta-analysis of risk factors for ICI and added the most important risk factors (renal replacement therapy and previous antimicrobial therapy) to the elements of the CCI [36]. The meta-analysis also revealed that previous abdominal surgery in particular, rather than any previous surgery, is associated with a high risk of ICI. Multifocal *Candida* colonization is another known risk factor for developing ICI [37]. However, we did not choose colonization as an inclusion criterion. Surveillance cultures for *Candida* colonization are not taken in most of the study centers. In addition, microbiological results are usually not available at the onset of sepsis when study enrolment must be decided.

The SEPSIS-3 definition was published during the development of this study [21]. At this stage, we decided not to change the inclusion criteria to the Sepsis-3 definitions, since all sample size calculations relied on studies that were based on the consensus criteria of the American College of Chest Physicians and Society of Critical Care Medicine [38]. We did, however, abandon the requirement for SIRS to qualify for enrolment. The development of acute organ dysfunction as a cause of infection is the main pathophysiological step that determines the prognosis of the patient. SIRS does not affect the outcome and does not predict the development of severe sepsis or septic shock [39–41].

The sample size calculation for this study was difficult since ICI often remains unrecognized. ICI is mainly diagnosed as candidemia but deep-seated candidiasis is commonly not accompanied by positive blood cultures [5]. Thus, many assumptions had to be made. We have chosen the approach of the EMPIRICUS trial [42] to estimate the number of patients who may benefit from early empiric antifungal therapy and replaced the estimates with results from more recent trials. A recent retrospective study on 198 patients with elevated CCI confirmed our estimates. Of these patients, 31.8% had elevated BDG serum concentrations of which 74.6% had proven candidemia. All episodes of candidemia occurred in the elevated BDG group [43]. This aligns quite well with our estimate that 28% of the patients at risk are assumed to develop ICI.

The strengths of this study include the multicenter, randomized, controlled design. The trial is undertaken according to good clinical practice guidelines. The study interventions are based on guideline recommendations, therefore they follow closely clinical practice. The participating hospitals represent standard care in Germany. Thus, the results of this study are generalizable to similar health-care settings.

Our trial has limitations. The current version of the Fungitell® assay does not allow measurement of serum BDG concentrations in each local laboratory. A central laboratory had to be established to ensure the uniform quality of the BDG results. However, such a setup will not always allow us to keep the time to result below 24 h, which is the desired time frame for keeping mortality rates as low as possible [7]. Sample are shipped via a courier service to minimize delays. Certain hours of enrolment are ruled out individually for each center, if timely shipping cannot be guaranteed by the courier service. The time window for study inclusion had to be increased from 12 to 24 h because of a high number of screening failures. This may further increase the time to antifungal therapy in the BDG group and therefore, might reduce the difference in primary and secondary outcomes between the two groups. However, the recruitment rate before the amendment was too low to allow completion of the trial in due time. The study has a risk of performance and detection bias with respect to diagnosing and treating ICI, since the trial design does not allow blinding. The lack of blinding may trigger the treating physician to focus more on ICI due to the BDG reporting in the BDG group. In the control group, however, ICI diagnosis and therapy is left to the discretion of the physician without reminders by the study protocol. Training on the European guidelines regarding the management of ICIs [10], which all centers agreed to follow, tries to minimize this issue. We measure BDG only at the onset of sepsis. However, ICI might develop later during the course of sepsis. Although it might be helpful for patient management to have BDG guidance available during the whole ICU stay, this was beyond the financial scope of the study.

This is the first randomized controlled study to investigate whether BDG serum concentrations can guide a physician in supplementing the empiric antimicrobial therapy with an antifungal in critically ill patients with severe sepsis or septic shock. This clinical study will generate a solid evidence base for health-care providers and authors of guidelines for BDG in critically ill patients.

### Trial status

The study opened for recruitment on 15 September 2016. As of 31 July 2018, 176 patients (78 patients before the amendment and 98 patients after the amendment) have been enrolled into the study. Completion of recruitment is expected in September 2019.

## Abbreviations

BDG: (1,3)-β-D-glucan
CCI: *Candida* colonization index
DSMB: Data and safety monitoring board
eCRF: Electronic case report form
EDTA: Ethylenediaminetetraacetic acid
ICI: Invasive *Candida* infection
ITS: Internal transcribed spacer region
ICU: Intensive care unit
NRZMyk: National Reference Center for Invasive Mycoses
PCR: Polymerase chain reaction
SOFA: Sequential organ failure assessment
